# Coolings

**Published:** 1859-06

**Authors:** 


### COOLINGS.

To make water almost ice cold, keep it in an earthen pitcher, unglazed, wrapped around with several folds of coarse linen, or cotton cloth, kept wet all the time. The evaporation from the cloth abstracts the heat from within, and leaves the water as cold as it ought to be drank in summer, consistent with safety and health.

Cooling rooms: the least troublesome plan is to hoist the windows and open the doors at daylight, and at eight or nine o'clock, close them, especially the external windows and shutters, if there be any, except to admit barely necessary light.

Churches may be kept delightfully cool in the same way, and thus greatly add to the comfort of public worship, leaving the windows open, but the lattice shutters closed, on the north side of the house, which will secure a thorough ventilation.

Still greater coolness may be produced by having a large heavy cotton or linen sheet hung near each open window or door, and kept constantly wet, the evaporation produces a vacuum, and a continual draft of air is the result. In India and other eastern countries, common matting is used; long grass plaited answers a good purpose. In Germany, a broad vessel or pan is kept in the room, nearly filled with water, the pan, not the room, the surface of the water being covered with green leaves.

To have delightfully hard butter in summer, without ice, the plan recommended by that excellent and useful publication the Scientific American, a year ago, is a good one. Put a trivet or any open flat thing with legs, in a saucer; put on this trivet, the plate of butter, and fill the saucer with water; turn a common flower pot upside down over the butter, so that its edge shall be within the saucer, and under the water. Plug the hole of the flower pot with a cork, then drench the flower pot with water, set it in a cool place until morning; or if done at breakfast, the butter will be very hard by supper time. How many of our city boarding school girls, who have been learning philosophy, astronomy, syntax and prosody for years, can, of their ownselves, write us an explanation, within a month.

To keep the body cool in summer, it is best to eat no meat, or flesh, or fish, at least not oftener than once a day, and that in the cool of the morning; making a breakfast dessert of berries of some kind. Dinner, light soup with bread; then vegetables, rice, samp, corn, cracked wheat; dinner dessert of fruits and berries. in their natural state, fresh, ripe and perfect. Touch nothing later than dinner; taking nothing at all at supper, but a piece of cold bread and butter, and a single cup of some hot drink, or in place of these, a saucer of ripe berries, without sugar, milk, cream or any thing else, not even a glass of water, or any other liquid, for an hour after.

To keep the head cool, especially of those who live by their wits, such as lawyers, doctors, editors, authors, and other gentlemen of industry, it is best to rise early enough to be dressed and ready for study, as soon as it is sufficiently light to use the eyes easily without artificial aid, having retired the evening before, early enough to have allowed full seven hours for sound sleep; then study for about two hours; next make a breakfast of a piece of cold bread and butter, an egg, and a cup of hot drink, nothing more; then resume study until ten, not to be renewed until next morning; allowing no interruption whatever, until the time for study ceases, except to have the breakfast brought in. The reason of this is, the brain is recuperated by sleep ; hence its energies are greatest, freshest, purest, in all men, without exception, immediately after a night' ssleep, and every moment of thought, diminishes the amount of brain power, as certainly as an open spiggot diminishes the amount of liquid within. Nature may be thwarted, and her plans wrested from her ; and habit or stimulation may make it more agreeable to some to do their studying at night, but it is a perversion of the natural order of things, and such persons will be either prematurely disabled, or their writings will be contrary to the right and the true. As the brain is more vigorous in the morning, so is the body, and vigor of both must give vigor of thought and expression, that is, if the head has any thing inside.

